# *In vivo* imaging to monitor differentiation and therapeutic effects of transplanted mesenchymal stem cells in myocardial infarction

**DOI:** 10.1038/s41598-017-06571-8

**Published:** 2017-07-24

**Authors:** Zhijun Pei, Jing Zeng, Yafeng Song, Yan Gao, Ruimin Wu, Yijia Chen, Fuyan Li, Wei Li, Hong Zhou, Yi Yang

**Affiliations:** 1 Department of PET Center and Institute of Anesthesiology and Pain, Taihe Hospital, Hubei University of Medicine, Shiyan, Hubei 442000 China; 2 Department of Infection Control, Taihe Hospital, Hubei University of Medicine, Shiyan, Hubei 442000 China

## Abstract

Here, we used a noninvasive multimodality imaging approach to monitor differentiation of transplanted bone marrow mesenchymal stem cells (BMSCs) and recovery of cardiac function in an *in vivo* model of myocardial infarction (MI). We established a rat MI model by coronary artery ligation. Ninety rats were randomly assigned into four groups: sham-operated, MI model, and α-MHC-HSV1-tk-transfected or un-transfected BMSCs-treated MI model. We used ^18^F-Fluro-deoxyglucose (^18^F-FDG) positron emission tomography (PET) to monitor recovery of cardiac function, and ^18^F-FHBG PET/CT imaging to monitor transplanted BMSCs differentiation 24 h after ^18^F-FDG imaging. The uptake of ^18^F-FDG at 3, 16, 30 and 45 days after BMSCs injection was 0.39 ± 0.03, 0.57 ± 0.05, 0.59 ± 0.04, and 0.71 ± 0.05% ID/g, respectively. Uptake of ^18^F-FHBG increased significantly in large areas in the BMSCs-treated group over time. *Ex vivo* experiments indicated that expression of the cardiomyocyte markers GATA-4 and cardiac troponin I markedly increased in the BMSCs-treated group. Additionally, immunohistochemistry revealed that HSV-tk-labelled BMSCs-derived cells were positive for cardiac troponin I. Multimodal imaging systems combining an α-MHC-HSV1-tk/^18^F-FHBG reporter gene and ^18^F-FDG metabolism imaging could be used to track differentiation of transplanted BMSCs and recovery of cardiac function in MI.

## Introduction

Ischaemic heart disease is a serious threat to human health, and stem cell transplantation may be an effective treatment^1, 2^. There has been intense interest in developing treatments to repair the damaged heart tissue and restore cardiac function. Recent studies have demonstrated that BMSCs exhibit self-renewal and multipotency, and they are considered ideal progenitor cells for stem cell transplantation^3–5^. They are easily obtained and cultured, and express exogenous genes efficiently. For treatment of serious heart conditions such as MI, numerous basic and clinical studies indicate that transplantation of BMSCs into the vicinity of the damaged myocardium through the coronary artery increases angiogenesis and blood supply to the heart, reduces scar formation and fibrosis, promotes myocardial tissue repair or regeneration, and improves heart function^6–8^. Furthermore, vascular endothelial growth factor (VEGF) improves the survival of MSCs in ischemic regions. In animal models and phase I clinical trials, VEGF therapy significantly improved myocardial perfusion and function^9^.

Monitoring the survival and migration of transplanted stem cells via noninvasive means *in vivo* is crucial for the success of stem cell transplantation and the treatment of ischaemic heart disease. Over the past decade, there have been considerable advances in imaging technologies for tracking stem cells^10, 11^ and for visualising targeted cellular processes at the molecular or genetic level in whole-body studies of living subjects. In particular, reporter gene imaging has been developed to allow evaluation of biological processes in transplanted stem cells at the cellular and molecular levels^12, 13^. We previously demonstrated that a fusion reporter gene of herpes simplex virus type 1 thymidine kinase (HSV1-tk), eGFP, and firefly luciferase can be used for *in vivo* monitoring of transplanted BMSCs^14^. Conventional reporter gene imaging techniques have been used to monitor and track BMSCs and can provide information on important features of cellular implants, such as cell viability, and migration^15^. However, the lack of convincing therapeutic success of BMSCs transplantation can be partly attributed to the inefficient monitoring of differentiation and recovery of cardiac function *in vivo*, which plays a key role in the rapid assessment of therapy procedures in preclinical models.

We hypothesised that by placing a PET reporter gene under the control of the cardiac-specific α-myosin heavy chain (α-MHC) promoter, the activity of which is enhanced during cardiac differentiation, we would be able to monitor BMSCs in a rat model of MI by dynamically imaging the linked reporter gene (HSV1-tk). Furthermore, we sought to monitor the recovery of cardiac function after treatment with BMSCs and VEGF by ^18^F-FDG PET imaging. If the transfected BMSCs successfully differentiated into myocardial cells and restored myocardial function, continuous dynamic monitoring would detect FDG uptake in the BMSCs-treated rat MI model. Based on our previous study of PET imaging with multimodality reporter genes in a rat MI model^14^, we constructed a reporter gene under the control of the cardiac-specific α-MHC promoter. We then generated stable α-MHC-HSV1-tk-expressing cell lines by lentiviral transduction of BMSCs. Taken together, our findings establish a multimodality imaging approach for monitoring differentiation changes and therapeutic effects of transplanted BMSCs and VEGF for the treatment of MI *in vivo*.

## Results

### Animal models and general conditions

**Figure 1:**
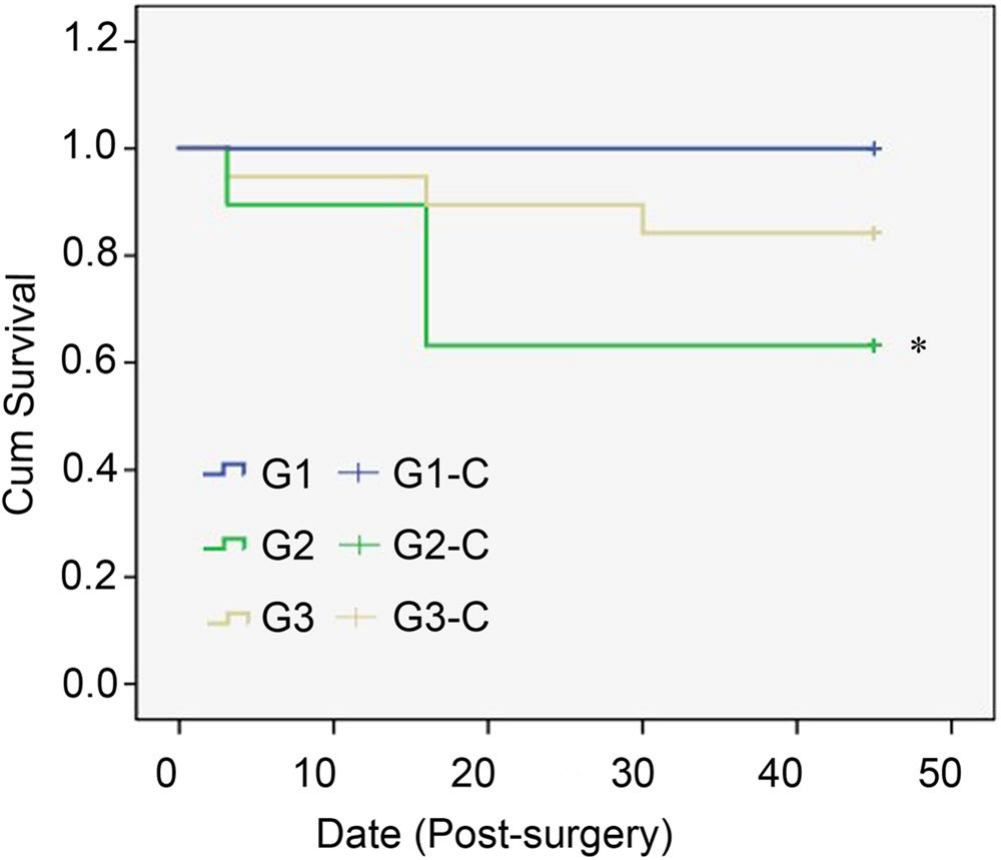
Survival curves for the sham-operated group (G1), the MI model (G2) and BMSC-treated MI group (G3).

90 rats were assigned into four groups before surgery: sham-operated (G1), MI model (G2), and α-MHC-HSV1-tk-transfected BMSCs-treated MI model (G3), BMSCs-treated MI model (G4). There were 1, 8, 9 and 11 deaths in each group among the rats subjected to surgery. A total of 29 died of intraoperative ventricular fibrillation, cardiac arrest, or unknown causes, 19 rats had survived in G2, G3 and G4 groups each. All the rats in G1 survived during the rest of the study period. In contrast, deaths occurred in G2, G3 and G4, particularly in the period from 3 days to 1 week (Fig. 1). However, the overall survival rate was significantly higher in G3 (84.2%) than in G2 (52.6%) 46 days after surgery (x^2^ = 4.385, p = 0.036).

### Multimodality imaging in rats

**Figure 2:**
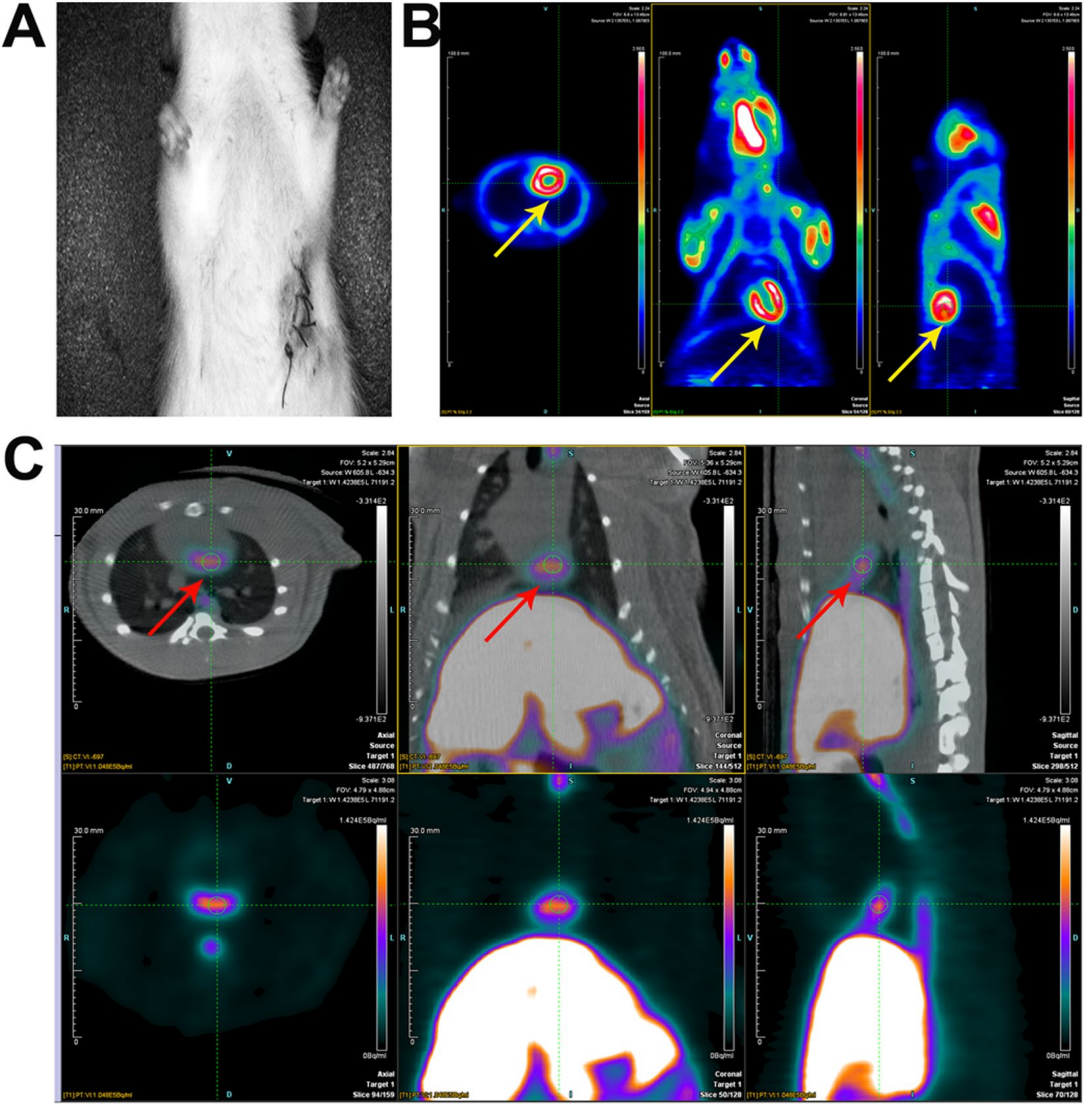
Multimodality molecular imaging to monitor transplanted BMSCs in a rat model of MI. Yellow and red arrows indicate the site of transplanted BMSCs. Transverse, coronal and sagittal images of the MI surgical site (**A**), ^18^F-FDG PET images (**B**), and ^18^F-FHBG PET/CT fusion images (**C**) of the transplant region.

To assess changes in myocardial perfusion, transverse, coronal and sagittal ^18^F-FDG micro-PET images of myocardial glucose metabolism were acquired before and after the BMSCs treatment period. Dynamic data were used for visualisation of regional ^18^F-FDG uptake. Figure 2B shows a representative rat from the transfected-BMSCs-treated MI model (G3). Myocardial radioactivity appeared homogeneous and normal 45 days after BMSCs injection. In the same manner, 9-(4-[18] F-fluoro-3-[hydroxymethyl] butyl) guanine (^18^F-FHBG) PET/computed tomography (CT) images were obtained to monitor the labelled transplanted BMSCs in G3. The fusion image in Fig. 2C shows obvious ^18^F-FHBG uptake at the injection site in the left upper forelimb of the rats.

### Changes in relative myocardial glucose metabolism after treatment

**Figure 3:**
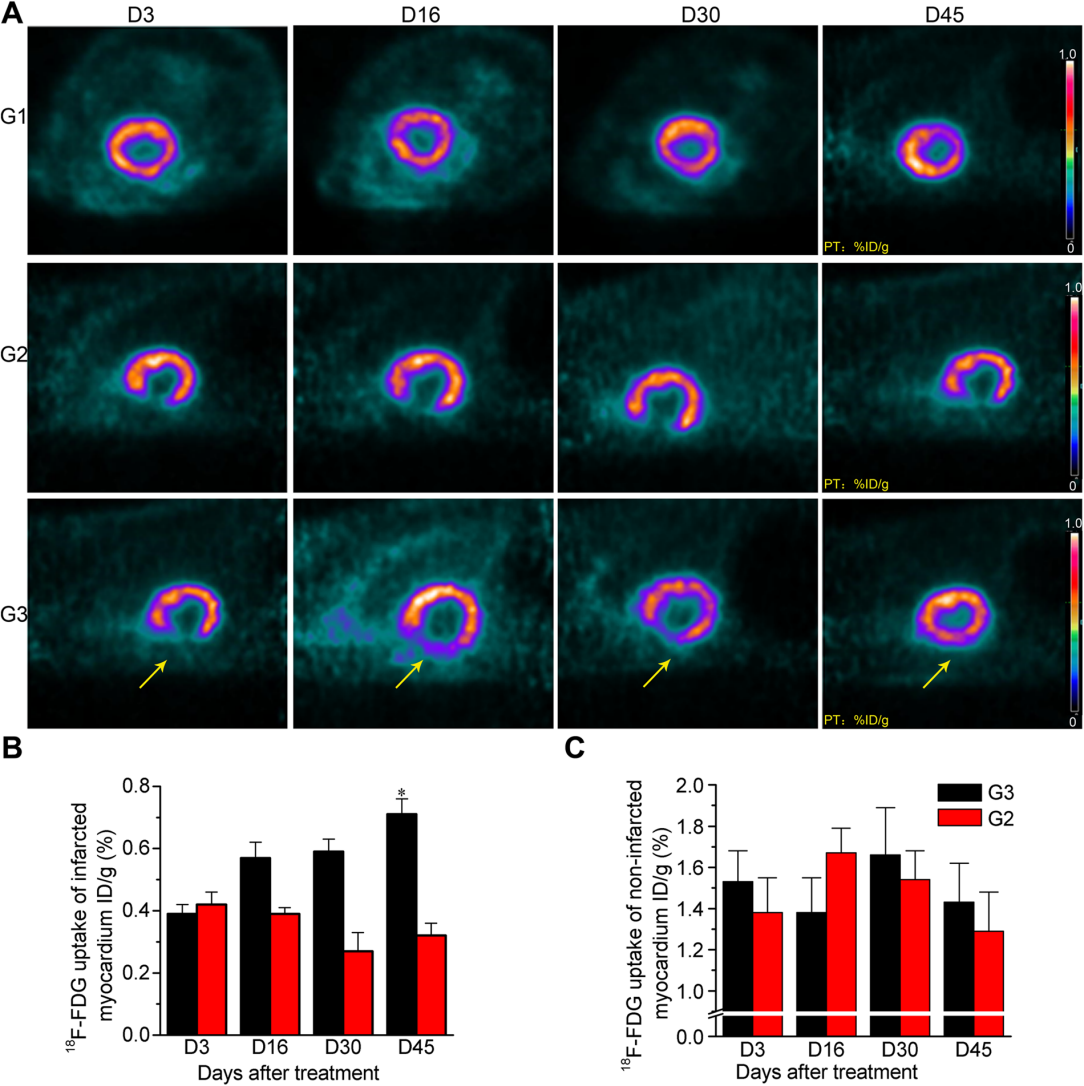
Myocardial activity measured using ^18^F-FDG microPET imaging (**A**), and ^18^F-FDG uptake in infarcted (**B**) and non-infarcted (**C**) myocardium of the MI model (G2) and transfected-BMSC-treated MI model (G3) groups during the therapy phase. Yellow arrows indicate transplanted BMSCs *P < 0.05.

Recovery in cardiac metabolic function was assessed by analysing FDG uptake in infarcted and normal myocardium in all rats (Fig. 3A). Continuous dynamic monitoring revealed varying degrees of reduction of radioactivity distribution in the myocardium of G2 and G3 during the early stage of the monitoring period. The distribution of radioactivity in the MI area gradually recovered in G2 Over time but did not recover in G2. No major morphological differences were observed between G3 and G1 after 45 days. Next, quantitative analysis of the relative regional activity was performed in preselected regions of interest (ROIs; infarcted and non-infarcted myocardium). As shown in Fig. 3B, myocardial glucose metabolism in the infarcted myocardium increased slightly over time in G3, and was significantly higher at day 45 than that in G2 (p < 0.05). ^18^F-FDG uptake at 3, 16, 30 and 45 days was 0.39 ± 0.03, 0.57 ± 0.05, 0.59 ± 0.04 and 0.71 ± 0.05% ID/g, respectively, in G3, compared with 0.42 ± 0.03, 0.39 ± 0.05, 0.27 ± 0.0, and 0.32 ± 0.05% ID/g in G2, respectively. There were no significant differences in glucose metabolic observed in non-infarcted myocardium between G2 and G3 (Fig. 3C).

### Reporter gene imaging for BMSCs differentiation monitoring

**Figure 4:**
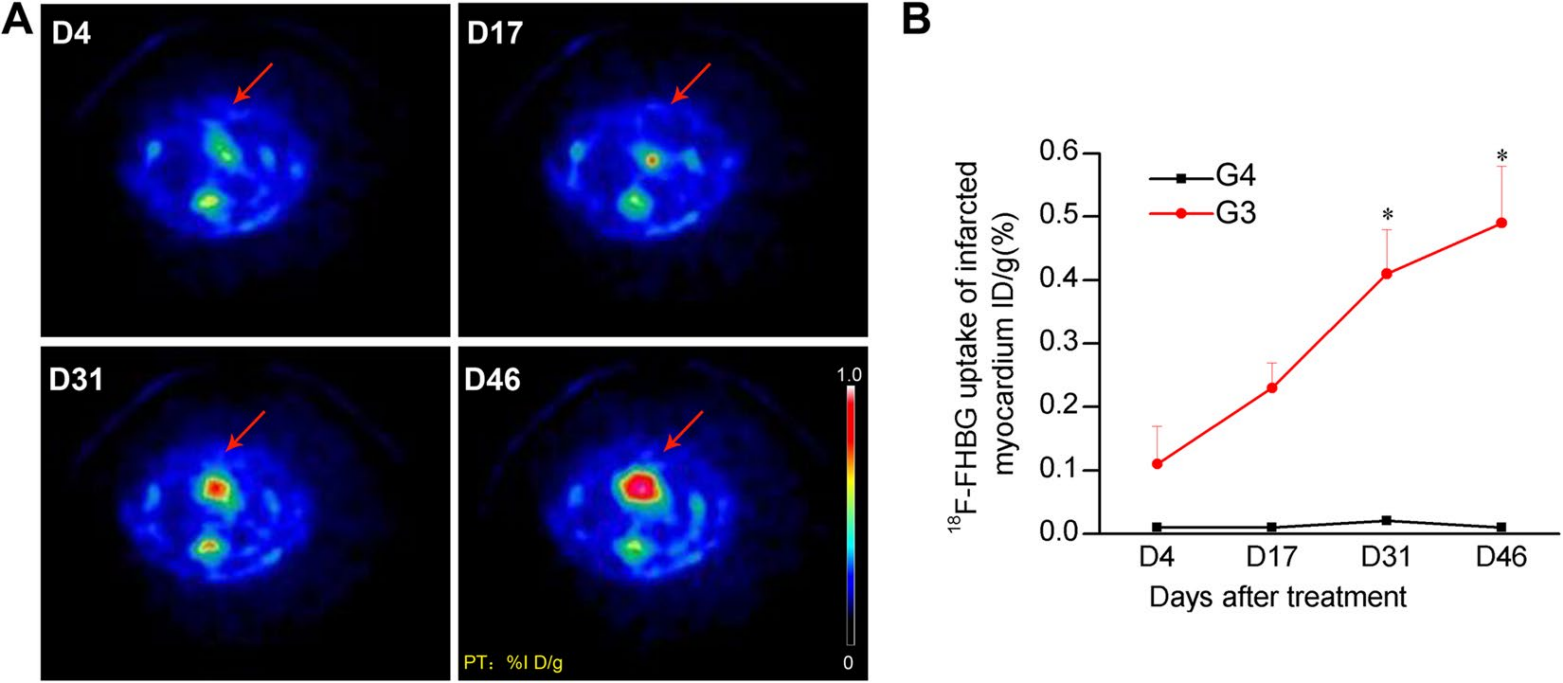
^18^F-FHBG imaging and uptake analysis of BMSCs in a rat model of MI. (**A**) Transverse ^18^F-FHBG microPET/CT images of BMSCs (red arrows) in the transplant region 4, 17, 31, and 46 days after treatment. (**B**) Quantification of ^18^F-FHBG uptake in infarcted myocardium (% ID/g). G4, untransfected-BMSC-treated MI model. *P < 0.05 vs. G3, transfected-BMSC-treated MI model.

Selective uptake and retention of ^18^F-FHBG by BMSCs transduced with α-MHC-HSV1-tk (G3) are shown in Fig. 4A. The uptake at 4, 17, 31 and 46 days was 0.11 ± 0.06, 0.23 ± 0.04, 0.41 ± 0.07, and 0.49 ± 0.09% ID/g, respectively. Over time, ^18^F-FHBG uptake increased significantly in large parts of the ROIs in G3 (Fig. 4B). However, ^18^F-FHBG uptake in the MI with non-transfected BMSCs group (G4) did not change compared with the baseline. To better understand the mechanism by which stem cell therapy promotes functional recovery, we examined cell-surface marker expression on myocardial muscles by immunohistochemistry.

### *Ex vivo* cardiac differentiation of BMSCs

**Figure 5:**
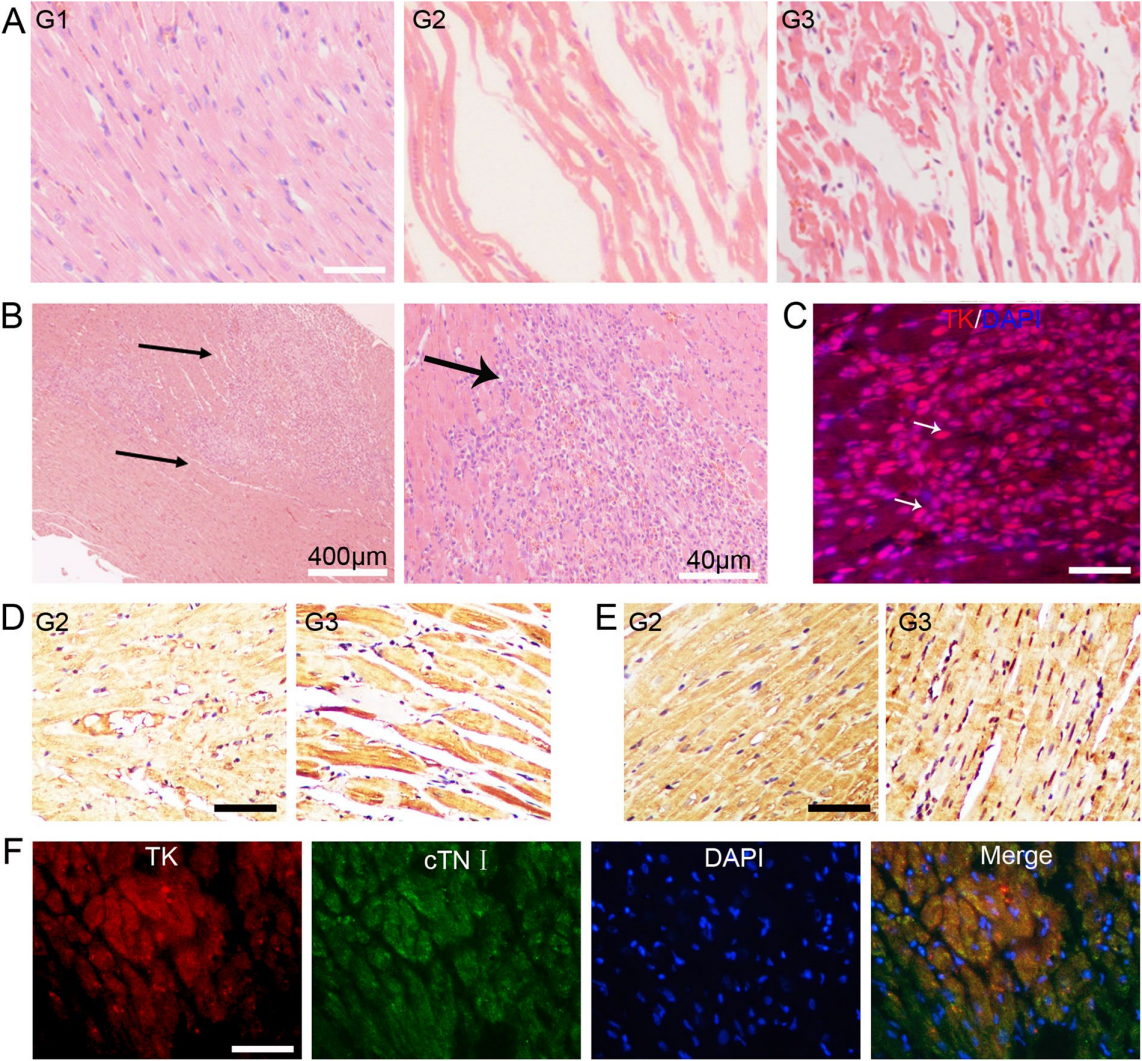
Characterisation of the MI model and *ex vivo* analysis of rat myocardial tissue 45 days after MI. (**A**) Haematoxylin and eosin staining of myocardium from the sham-operated (G1), MI model (G2), and transfected-BMSC-treated MI model (G3). Scale bar, 50 μm. (**B**) Haematoxylin and eosin staining of heart tissue sections from the G3 group. Arrows indicate BMSCs. (**C**) HSV1-tk (red) and DAPI (blue) immunohistochemical staining of rat heart tissue from the G3 4 days after treatment. Arrows indicate overlapping (i.e. positive) staining of transplanted labelled BMSCs. Scale bar, 30 μm. (**D**) Immunostaining for GATA-4 and cardiac troponin I (cTnI) (**E**) in myocardium derived from α-MHC-induced differentiation of BMSCs. Scale bar, 50 μm. (**F**) Immunocytochemical analysis of HSV1-tk (red) and the cardiomyocyte-specific marker cTnI (green), and DAPI staining (blue), and their colocalisation in heart tissue from the G3 45 days after transplantation. Scale bar, 50 μm.

The healthy myocardium took the form of regular bundles of fibres. Conversely, the fibres of the infarcted myocardium were swollen and disordered, and there were vacuoles and sometimes even fractures present (Fig. 5A). As shown in Fig. 5B, BMSCs were located in the gaps between the fibre bundles shortly after transplantation. We then analysed the expression of cardiac precursor-specific and cardiomyocyte-specific proteins by immunohistochemistry with anti-HSV1-tk, anti-GATA-4 and anti-cardiac troponin I (cTnI) antibodies. Positive staining for these markers was detected in myocardial cells in G3: When the α-MHC-HSV1-tk-transfected BMSCs had differentiated, HSV1-tk was detected in the myocardial tissues (Fig. 5C). However, no obvious staining was detected in G1 or G2. Furthermore, the immunohistochemistry staining indicated that expression levels of the cardiac transcription factor GATA-4 were clearly higher in the G3 (Fig. 5D). Additionally, the cardiac muscle-specific marker cTnI was markedly upregulated, and the cells that stained positive for cTnI exhibited better-organised cross-striated myofilaments in the G3 (Fig. 5E). In G3, colocalisation of HSV-tk (red) and the cardiac marker cTnI (green) was observed on immunohistochemically-stained tissue slices (Fig. 5F). The results indicate that the transplanted BMSCs differentiated to cardiomyocytes after α-MHC-HSV1-tk-transfected-BMSCs injections.

## Discussion

Previous investigations have provided strong evidence to support transplantation of BMSCs with VEGF to be an effective treatment for ischemic heart disease. However, stem cells are transplanted into the ischemic area where various factors may cause cell death, such as the host inflammatory response^16^, apoptosis^17, 18^, and cytotoxins^19^. The viability of transplanted BMSCs is important during the inception phase of stem cell transplantation for MI treatment. After the stem cells have migrated to the target tissue, their differentiation to mature functional cells is critical for successful therapy^20, 21^. Another essential feature of stem cell therapies for heart conditions is the ability of the stem cells to replace necrotic cardiomyocytes. Monitoring the activity of transplanted BMSCs *in vivo*, especially during the early stage of transplantation, is crucial to allow prediction of their effects and adjustment of the treatment regime to optimise the therapeutic effects. Previous investigations have demonstrated that stem cell viability and migration can be visualised and tracked using noninvasive molecular imaging, with reporter gene techniques proving especially useful^22, 23^. However, further studies are required to monitor differentiation *in vivo*.

Transplantation of BMSCs with a reporter gene is a good combination^24^. In this study, we developed a cardiomyocyte-specific HSV1-tk reporter gene expression system using the α-MHC gene promoter, a specific regulator. In this system, HSV1-tk is expressed only when the BMSCs differentiate directly into myocardial cells, thus allowing stem cell differentiation to be monitored *in vivo*. At the same time, the cells can be tracked by ^18^F-FHBG PET imaging. Our results demonstrate the feasibility of this approach, α-MHC-HSV1-tk/^18^F-FHBG microPET imaging enabled monitoring and quantitative analysis of the differentiation of BMSCs after transplantation. In the present study, we clearly showed that α-MHC enhanced cardiac differentiation of BMSCs, evidenced by the fact that expression of the cardiac transcription factor GATA-4 and the cardiac muscle-specific marker cTnI were markedly enhanced in the lablled-BMSCs group. Moreover, the HSV-tk-labelled BMSC-derived cells in the G3 were also uniquely positive for these specific cardiac markers (Fig. 5F). The mechanism by which transplanted BMSCs in the myocardium only give rise to cardiomyocytes remains to be further investigated.

Both myocardial glucose metabolism imaging and myocardial fatty acid metabolism imaging are important for allowing efficient, noninvasive, and rapid assessments of myocardial activity and transgene expression in preclinical models. Currently, radiolabelled probes such as ^11^C-palmitic acid are used in conjunction with PET/CT to assess myocardial beta-oxidation and metabolism during fasting. Unfortunately, the imaging quality of ^11^C-labelled probes is relatively poor, and the clinical applications of this approach are limited because of the rapid *in vivo* degradation and short half-life (approximately 20 min) of ^11^C^25^. Elevated insulin and blood glucose levels and decreased free fatty acid (FFA) levels lead to a rise in myocardial glucose consumption, making glucose a primary source of energy in the myocardium during ^18^F-FDG myocardial glucose metabolism imaging on non-fasted rats with high plasma levels of glucose and insulin. Conversely, beta-oxidation is the major energy source for cardiomyocytes during fasting, because an increase in FFA levels and a decrease in insulin and glucose levels shifts myocardial energy consumption away from glucose and toward FFA. Therefore, we believed that ^18^F-FDG PET myocardial glucose metabolism imaging showed better selectivity in assessments of myocardial activity in our model than did ^11^C-palmitic acid. We applied the technique in combination with a reporter gene to allow us to evaluate myocardial activity by ^18^F-FDG microPET imaging^26^. Using continuous dynamic monitoring, no FDG uptake signal was detectable in the infarcted area in G2 group 45 days after MI because of myocardial cell necrosis. Conversely, the radioactivity gradually became distributed through the infarcted area in the G3 group over time. Recovery of cardiac metabolic function demonstrated that the BMSCs transplanted into the infarcted tissue differentiated into cardiomyocytes and restored the function of myocardium. Such a noninvasive, rapid, and continuous method for monitoring BMSCs in real-time may provide technical support for further improvement of stem cell transplantation technologies.

Each of the aforementioned imaging technologies has unique advantages and disadvantages; our study presents an optimal imaging modality. Despite its specificity and efficacy, the HSV1-tk/^18^F-FHBG microPET system had a low capacity for localised diagnosis, and the use of additional microPET/CT to monitor the location would be of great value. However, location is not essential for ^18^F-FDG microPET imaging of myocardial metabolism. Therefore, we established ^18^F-FDG PET imaging without CT positioning to reduce the duration of image acquisition. Furthermore, we monitored myocardial activity using ^18^F-FHBG and ^18^F-FDG microPET imaging on separate days, because the two positron imaging agents have different properties, so their interaction should be avoided. In the present study, we clearly showed by immunohistochemistry that α-MHC-HSV1-tk enhanced BMSC differentiation and recovery of cardiac function. Although these experiments reveal a new multimodality imaging strategy, the study also has some limitations. Extensive ongoing progress in stem cell therapy and molecular imaging technologies means that stem cell transplantation in conjunction with cytokines^27–29^ and therapeutic genes^30, 31^ will play an increasingly important role in therapies for many diseases.

In conclusion, multimodal imaging systems combining an α-MHC-HSV1-tk/^18^F-FHBG reporter gene and ^18^F-FDG metabolism imaging can be used to monitor the viability, migration and differentiation of transplanted BMSCs and the recovery of cardiac function in a rat model of MI. Therefore, changes in cardiomyocyte activity and transplanted BMSCs *in vivo* can be visualised dynamically.

## Methods

### Virus construction and cell culture

The CMV promoter of the pMOD-CMV-HSV1-tk (InvivoGen, CA) backbone plasmid was replaced with the cardiac-specific promoter of the α-MHC gene. The reconstructed pMOD-α-MHC-HSV1-tk plasmid was repackaged into a recombinant lentivirus (lenti-α-MHC-HSV1-tk). Virus was amplified and purified, and the titre was 1.26 × 10^8^ TU/ml.

BMSCs were isolated from the femur and tibia of a healthy 4-week-old Sprague–Dawley (SD) rat supplied by the Experimental Animal Center of Hubei University of Medicine. The bone marrow cells were flushed out with Dulbecco’s modified Eagle’s medium/F12 medium containing 15% foetal bovine serum and then seeded in six-well plates. Passage 3–5 BMSCs were infected with lenti-α-MHC-HSV1-tk. The resultant stable cell line expressing α-MHC-HSV1-tk was characterised by flow cytometry and incubated at 37 °C in a humidified incubator with 5% CO_2_.

### Establishment and characterisation of the MI rat model

Specific-pathogen-free SD rats were provided with food and water ad libitum. All animal procedures were approved by the Laboratory Animals Ethics Committee of Hubei University of Medicine. All procedures involving experimental animals were performed in in strict compliance with local animal welfare laws, guidelines and policies. The MI model was established according to the method of Fisbein *et al*.^32^ and our previous studies^14, 33^. The animals were fasted for 12 h then anesthetised with 2% isoflurane by inhalation. The rats were subjected to a left lateral thoracotomy and pericardectomy (Fig. 2A), then the left coronary artery (LCA) was identified and gently ligated with a 6-0 prolene suture. All rats received 40,000 U penicillin (0.2 ml) by intramuscular injection within 6 h of surgery and were monitored during the next day. A total of SD rats (200 ± 25 g) were randomly assigned into four groups: a sham surgery group (G1, n = 5), in which the LCA was located and threaded, but not ligated; an MI model group (G2, n = 27), in which the LCA was ligated; a treatment group (G3, n = 28), in which approximately 3 × 10^6^ stably α-MHC-HSV1-tk-transduced BMSCs and 100 ng/ml VEGF in 50 µl PBS were injected at ligation sites at a depth of 2 mm; and a control treatment group (G4, n = 30), injected with 3 × 10^6^ BMSCs and 100 ng/ml VEGF at ligation sites.

### ^18^F-FDG and ^18^F-FHBG microPET/CT imaging

A Siemens Inveon™ Acquisition Workplace (Inveon mPET/CT; Siemens Preclinical Solution, Knoxville, TN) was used for microPET/CT imaging. ^18^F-FDG and ^18^F-FHBG were automatically synthesised using a Multi-functional Composite Module (F300Ek, Sumitomo Heavy Industries, Ltd., Japan). MicroPET imaging was performed for 10 min at 3, 16, 30 and 45 days after stem cell injection. All the rats were allowed ad libitum access to food and drinking water containing 0.5% glucose for at least 8 h prior to imaging to ensure high levels of plasma glucose and insulin at the time of ^18^F-FDG myocardial glucose metabolism imaging. Animals were anesthetised with 2% isoflurane, injected with ^18^F-FDG (300 µCi) through the tail vein, rested for 50 min to allow uptake of ^18^F-FDG to occur, and restrained in a scan bed to prevent any movement. CT images were acquired with 10 min static scanning followed by PET scanning in the heart area and whole body. To detect transplanted BMSCs, ^18^F-FHBG microPET/CT imaging was performed 24 h after ^18^F-FDG microPET imaging. The standard ordered-subset expectation maximisation method was used for microPET image reconstruction. CT images were used for both attenuation correction of emission data and image fusion.

### PET image analysis and statistical analysis

Static PET images of rats were acquired in three-dimensional mode and reconstructed iteratively twice using ordered-subset expectation maximisation (OSEM). ^18^F-FDG and ^18^F-FHBG uptake were measured in an ROI in the target region during the PET imaging analysis. The radioactivity in the ROI was measured as the mean percent injected dose/g (% ID/g), which normalises activity for body weight and injected activity. Relative regional activity was compared between groups and between pre- and post-treatment assessments using PMOD software V 3.2^34, 35^. We identified the whole heart area, infarcted area, and non-infarcted area in terms of relative activity in each group, to acquire the radioactivity value of each animal.

SPSS 22.0 software was used to analyse the data. All data are expressed as the mean ± standard deviation with the decay-corrected radioactivity concentration ROI value. Linear regression was used to analyse the linear relationship between two variables. A value of P < 0.05 was considered significant.

### Histological analyses

Rats were sacrificed by CO_2_ asphyxiation, and myocardial tissue was collected 45 days after surgery. The myocardial tissue was fixed in 4% paraformaldehyde, embedded in paraffin, sectioned at a thickness of 3 µm, hydrated, autoclaved, and then blocked with normal goat serum at room temperature for 10–15 min before being subjected to haematoxylin and eosin (HE) staining and immunohistochemistry. Sections of some G2 and G3 heart tissues were incubated with anti-HSV1-tk, anti-GATA-4 and anti-cTnI antibodies (Santa Cruz Biotechnology, Santa Cruz, CA) at 4 °C for 24 h, followed by a secondary antibody. The sections were then stained with diaminobenzidine (DAB), counterstained with haematoxylin or DAPI, dehydrated, mounted, and observed under an optical microscope.

## References

[1] Li W (2007). Bcl‐2 engineered MSCs inhibited apoptosis and improved heart function. Stem cells.

[2] Deuse T (2009). Hepatocyte growth factor or vascular endothelial growth factor gene transfer maximizes mesenchymal stem cell–based myocardial salvage after acute myocardial infarction. Circulation.

[3] Chong JJ (2014). Human embryonic-stem-cell-derived cardiomyocytes regenerate non-human primate hearts. Nature.

[4] Bianco P (2013). The meaning, the sense and the significance: translating the science of mesenchymal stem cells into medicine. Nature medicine.

[5] Fernandes S (2015). Comparison of Human Embryonic Stem Cell-Derived Cardiomyocytes, Cardiovascular Progenitors, and Bone Marrow Mononuclear Cells for Cardiac Repair. Stem cell reports.

[6] Templin C (2012). Transplantation and tracking of human-induced pluripotent stem cells in a pig model of myocardial infarction: assessment of cell survival, engraftment, and distribution by hybrid single photon emission computed tomography/computed tomography of sodium iodide symporter transgene expression. Circulation.

[7] Ye L (2014). Cardiac repair in a porcine model of acute myocardial infarction with human induced pluripotent stem cell-derived cardiovascular cells. Cell Stem Cell.

[8] Williams AR (2012). Enhanced effect of human cardiac stem cells and bone marrow mesenchymal stem cells to reduce infarct size and restore cardiac function after myocardial infarction. Circulation, Circulationaha..

[9] Madonna R (2015). Transplantation of adipose tissue mesenchymal cells conjugated with VEGF-releasing microcarriers promotes repair in murine myocardial infarction. Cardiovascular research.

[10] Higuchi T (2009). Combined reporter gene PET and iron oxide MRI for monitoring survival and localization of transplanted cells in the rat heart. Journal of Nuclear Medicine.

[11] Wang H (2009). Trafficking Mesenchymal Stem Cell Engraftment and Differentiation in Tumor‐Bearing Mice by Bioluminescence Imaging. Stem cells.

[12] Gyöngyösi M (2008). Serial noninvasive *in vivo* positron emission tomographic tracking of percutaneously intramyocardially injected autologous porcine mesenchymal stem cells modified for transgene reporter gene expression. Circulation: Cardiovascular Imaging.

[13] Qin C (2013). An *in vitro* and *in vivo* evaluation of a reporter gene/probe system hERL/(18)F-FES. PLoS One.

[14] Pei Z (2012). A multimodality reporter gene for monitoring transplanted stem cells. Nucl Med Biol.

[15] Kang JH, Chung JK (2008). Molecular-genetic imaging based on reporter gene expression. J Nucl Med.

[16] Shiba Y (2012). Human ES-cell-derived cardiomyocytes electrically couple and suppress arrhythmias in injured hearts. Nature.

[17] Zhu H, Sun A, Zou Y, Ge J (2014). Inducible Metabolic Adaptation Promotes Mesenchymal Stem Cell Therapy for Ischemia A Hypoxia-Induced and Glycogen-Based Energy Prestorage Strategy. Arteriosclerosis, thrombosis, and vascular biology.

[18] Abdelwahid, E. *et al*. Stem cell death and survival in heart regeneration and repair. *Apoptosis* 1–17 (2015). 10.1007/s10495-015-1203-4 PMC5200890 26687129

[19] Bao C (2010). Enhancement of the survival of engrafted mesenchymal stem cells in the ischemic heart by TNFR gene transfection This paper is one of a selection of papers published in this special issue entitled “Second International Symposium on Recent Advances in Basic, Clinical, and Social Medicine” and has undergone the Journal’s usual peer review process. Biochemistry and Cell Biology.

[20] Arthur, A., Zannettino, A. & Gronthos, S. Multipotential Mesenchymal Stromal/Stem Cells in Skeletal Tissue Repair. *Stem Cells and Bone Tissue* 82 (2013).

[21] Shen Y (2015). Comparison of magnetic intensities for mesenchymal stem cell targeting therapy on ischemic myocardial repair: high magnetic intensity improves cell retention but has no additional functional benefit. Cell transplantation.

[22] Nguyen PK, Riegler J, Wu JC (2014). Stem cell imaging: from bench to bedside. Cell stem cell.

[23] Cheung TH, Rando TA (2013). Molecular regulation of stem cell quiescence. Nature reviews Molecular cell biology.

[24] Rodriguez-Porcel, M., Wu, J. & Gambhir, S. *Molecular imaging of stem cells. StemBook [Internet]*. *2008*. 20614632

[25] David A (1999). Peterson, Janet F. Eary and Kenneth A. Krohn. Kinetic analysis of 2-[^11^C] thymidine PET imaging studies: validation studies. The Journal of Nuclear Medicine.

[26] Rodriguez-Porcel, M., Wu, J. C. & Gambhir, S. S. Molecular imaging of stem cells (2009). 20614632

[27] Cook G (2014). High-dose chemotherapy plus autologous stem-cell transplantation as consolidation therapy in patients with relapsed multiple myeloma after previous autologous stem-cell transplantation (NCRI Myeloma X Relapse [Intensive trial]): a randomised, open-label, phase 3 trial. The lancet oncology.

[28] Reikvam H (2013). Targeted anti-leukemic therapy as disease-stabilizing treatment for acute myeloid leukemia relapse after allogeneic stem cell transplantation: will it be possible to combine these strategies with retransplantation or donor lymphocyte infusions?. Current cancer drug targets.

[29] Hamshere, S. *et al*. Randomized trial of combination cytokine and adult autologous bone marrow progenitor cell administration in patients with non-ischaemic dilated cardiomyopathy: the REGENERATE-DCM clinical trial*. European heart journal*, ehv390 (2015). 10.1093/eurheartj/ehv390 PMC4654774 26333366

[30] Ong S-G (2014). Cross talk of combined gene and cell therapy in ischemic heart disease role of exosomal MicroRNA transfer. Circulation.

[31] Biffi A (2013). Lentiviral hematopoietic stem cell gene therapy benefits metachromatic leukodystrophy. Science.

[32] Gao E (2010). A novel and efficient model of coronary artery ligation and myocardial infarction in the mouse. Circ Res.

[33] Pei Z (2014). Multimodality molecular imaging to monitor transplanted stem cells for the treatment of ischemic heart disease. PLoS One.

[34] Xiong G (2012). Noninvasive image derived heart input function for CMRglc measurements in small animal slow infusion FDG PET studies. Physics in medicine and biology.

[35] Blankstein, R. & Miller, E. J. Quantifying FDG uptake to diagnose cardiac device infections: When and how should we do it? *Journal of Nuclear Cardiology* 1–3 (2015). 10.1007/s12350-015-0293-2 26494648

